# The Hitchhiker’s Guide to Untargeted Lipidomics Analysis: Practical Guidelines

**DOI:** 10.3390/metabo11110713

**Published:** 2021-10-20

**Authors:** Dmitrii Smirnov, Pavel Mazin, Maria Osetrova, Elena Stekolshchikova, Ekaterina Khrameeva

**Affiliations:** 1 Center of Life Sciences, Skolkovo Institute of Science and Technology, 121205 Moscow, Russia; Dmitrii.Smirnov@skoltech.ru; 2 Department of Life Sciences, Ben-Gurion University of the Negev, Beer Sheva 8410501, Israel; 3 V. Zelman Center for Neurobiology and Brain Restoration, Skolkovo Institute of Science and Technology, 121205 Moscow, Russia; P.Mazin@skoltech.ru (P.M.); Maria.Osetrova@skoltech.ru (M.O.); E.Stekolschikova@skoltech.ru (E.S.)

**Keywords:** lipidome, LC-MS, bioinformatics

## Abstract

Lipidomics is a newly emerged discipline involving the identification and quantification of thousands of lipids. As a part of the omics field, lipidomics has shown rapid growth both in the number of studies and in the size of lipidome datasets, thus, requiring specific and efficient data analysis approaches. This paper aims to provide guidelines for analyzing and interpreting lipidome data obtained using untargeted methods that rely on liquid chromatography coupled with mass spectrometry (LC-MS) to detect and measure the intensities of lipid compounds. We present a state-of-the-art untargeted LC-MS workflow for lipidomics, from study design to annotation of lipid features, focusing on practical, rather than theoretical, approaches for data analysis, and we outline possible applications of untargeted lipidomics for biological studies. We provide a detailed R notebook designed specifically for untargeted lipidome LC-MS data analysis, which is based on *xcms* software.

## 1. Introduction

Lipids represent the hydrophobic fraction of small biological molecules with a molecular weight below 1500 Da, known as metabolites [1]. Lipids play a crucial role in the cell, tissue, and organ physiology, acting not only as structural components of the membranes but also as signaling molecules and active members of various protein complexes. The significance of lipids is highlighted by a large number of studies and diseases involving the disruption of lipid metabolic enzymes and pathways, including neurological disorders, such as Alzheimer’s or Parkinson’s diseases, as well as diabetes and cancer [2,3,4,5,6,7,8].

**Figure 1:**
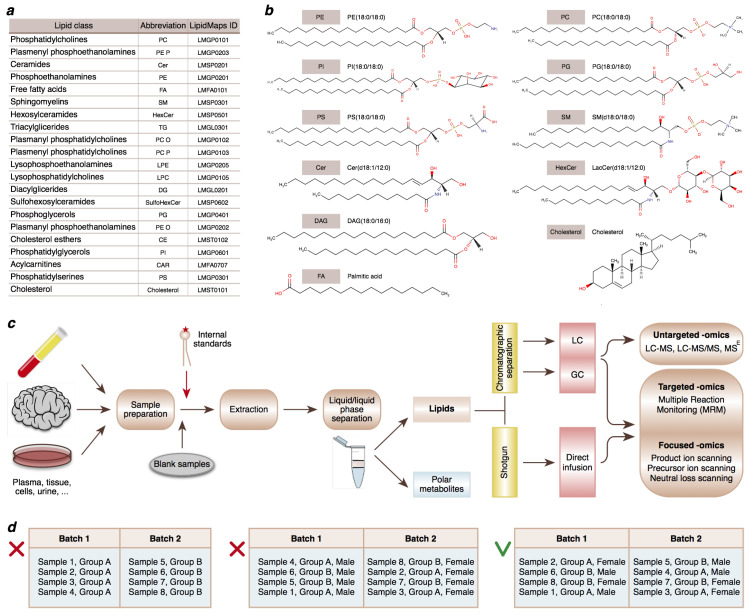
Lipid classes and lipidomics techniques discussed in this study. (**a**) Abbreviations and LIPIDMAPS identifications of lipid classes [13]. (**b**) Examples of prominent representatives of lipid subclasses [14]. (**c**) Experimental approaches that can be applied in lipidomics research. LC-MS and GC-MS are based on the separation of different lipid categories using extraction and chromatographic separation prior to mass analysis. Shotgun lipidomics omits chromatographic separation and analyzes all lipid classes together, directly infusing them into the mass spectrometer. (**d**) Balancing confounding factors between batches is an essential step of study design.

Over the last decade, the development of liquid chromatography coupled with mass spectrometry (LC-MS) has enabled comprehensive measurements of lipidome composition, yielding thousands of distinct MS peaks that represent individual lipid species. Such a large number of different lipid species arises from multiple combinations of fatty acids with base structures (Figure 1a,b). High-performance liquid chromatography (HPLC) covers many lipid classes, including sterols, glycerolipids, glycerophospholipids, sphingolipids, fatty acyls, and lipid headgroup derivatives.

Fatty acyls, containing a hydrocarbon chain that terminates with a carboxylic acid group, represent a diverse group of fundamental biological lipids that are commonly used as building blocks of more structurally complex lipids and precursors of biologically active lipids: prostaglandins, leukotrienes, and thromboxanes. Sterols, such as cholesterol and its derivatives, are important components of cellular membranes, along with glycerophospholipids: phosphatidylcholine, phosphatidylethanolamine, and phosphatidylserine. Glycerophospholipids, containing a phosphate group esterified to one of the glycerol hydroxyl groups, are also involved in the metabolism and cell signaling, and are especially abundant in neural tissues where alterations in their composition are linked to various neurological disorders.

The lipid composition of the myelin sheath is distinctive, made of a high amount of cholesterol and enriched in glycolipids, in the ratio of 40:40:20 (cholesterol, phospholipids, and glycolipids, respectively) compared to most biological membranes (25:65:10). In addition, some glycerophospholipids, i.e., phosphatidylinositols, can play the role of membrane-derived second messengers. Glycerolipids, including mono-, di-, and tri-substituted glycerols, function as an energy store and comprise the fat in animal tissues. Sphingolipids, containing a long-chain base as their core structure, represent another essential component of cellular membranes and include ceramides, sphingomyelins, and glycosphingolipids, which play important roles in signal transduction and cell recognition, especially in neural tissues.

While other experimental approaches can be applied in lipidomics research [7] (Figure 1c), in this study, we focus on the LC-MS method, which has become the analytical tool of choice for untargeted lipidomics because of its high sensitivity, convenient sample preparation, and broad coverage of lipid species [9]. We present a detailed LC-MS data analysis workflow designed specifically for untargeted lipidomics, which is based on the *xcms* software [10,11,12].

## 2. Experimental Design

### 2.1. Measurements of Lipidome Composition

LC-MS experimental workflow (Figure 1c) starts with sample preparation: homogenization of tissue samples or aliquoting samples of biological liquids. After this step, it is essential to add the isotope-labeled internal standards to the samples as early as possible to enable normalization for multiple potential sources of experimental biases at the data analysis stage.

Therefore, the extraction buffer is spiked with internal standards. The choice of standards depends on the lipids of interest and is selected according to the lipid class characteristic of the studied samples. After stratified randomization, lipid extraction is performed in batches of 48–96 samples. After every 23rd sample, a blank extraction sample is inserted, consisting of an empty tube without a tissue sample. These blank samples are essential for the analysis of the obtained LC-MS data because they serve as a baseline for filtering out peaks resulting from the extraction or other technical contamination. To achieve separation of the organic and aqueous phases, the samples are centrifuged, and the lipid fraction is selected.

To prepare quality control (QC) samples, an aliquot of each sample is additionally collected into a pooled sample. The mass spectra are then acquired for all samples processed in one sequence without interruption in positive and negative modes using an LC-MS system. QC samples are injected several times before initiating the run in order to condition the column, several times after each batch of samples, and after the completion of the run. QC samples are also injected after every ten samples to assess the instrument stability and analyte reproducibility. In addition, several blank samples are injected at the very beginning of the run and the very end of the run.

### 2.2. Study Design Considerations

The main limitation of LC-MS experiments is the small batch sizes compared to the total number of samples in large study cohorts. Typically, a batch of samples for LC-MS measurements includes 48–96 samples. At the same time, advanced studies tend to measure lipidome composition in thousands of samples because of the relatively small effect sizes compared to the technical and inter-individual variability associated with the confounding factors, such as sex, age, postmortem interval (PMI), smoking status, and others.

Moreover, despite adding internal standards and QC samples, the batch effect might still be visible even after thorough normalization. Thus, it is crucial to distribute samples among batches in a way that enables comparisons between groups of interest within the batch, and, most importantly, to avoid mixing the factor of interest with the batch covariate, as well as with the measurement order, because both of these confounding covariates might persist in the data after all normalizations and corrections.

In addition, it is essential to balance confounding factors between samples and controls and to randomize samples and controls in batches (Figure 1d). Technical replicates might be helpful for solving batch effect issues, but their use is not always practical in the case of large sample cohorts. Even without technical replicates, LC-MS runs can take several months as chromatographic separation takes about 30 min per sample, which, multiplied by 10,000 samples, results in 208 days.

### 2.3. Materials

This workflow is demonstrated on a test dataset obtained with a Reversed-Phase Bridged Ethyl Hybrid (BEH) C8 column reverse coupled to a Vanguard precolumn, using a Waters Acquity UPLC system and a heated electrospray ionization source in combination with a Bruker Impact II QTOF (quadrupole-Time-of-Flight) mass spectrometer. This untargeted lipidome LC-MS dataset consists of two sample groups (two samples per group) and a blank sample, thus, containing five samples in total.

### 2.4. Equipment

While many tools can be employed for LC–MS data analysis [10,11,12,15,16,17,18,19,20,21,22,23,24,25,26,27,28,29,30,31,32,33,34,35,36,37,38,39,40,41,42] (Table S1), this workflow is demonstrated with this suitable software combination:

- *ProteoWizard* cross-platform tool [43,44].
- *xcms* Bioconductor package (version 3.12.0) in the R environment [10,11,12].
- *IPO* Bioconductor R package (version 1.16.0) [45,46].
- *mixOmics* Bioconductor R package (version 6.14.1) [47].

## 3. Procedure of Data Analysis

### 3.1. Data Conversion

The LC-MS procedure results in an abundance of thousands of lipid species, measured as ion counts for a specific mass-to-charge ratio (m/z) and retention time (RT). While it is possible to store signals obtained by the MS instrument for all discrete m/z and RT values in the ‘profile data’ mode, the resulting files can be as large as 5 Gb per sample. To reduce this massive amount of data, MS instruments can export files in an alternative ‘centroid data’ mode, storing a single representative signal per peak and producing much smaller files, up to 400 Mb, without losing information relevant for further analysis.

**Figure 2:**
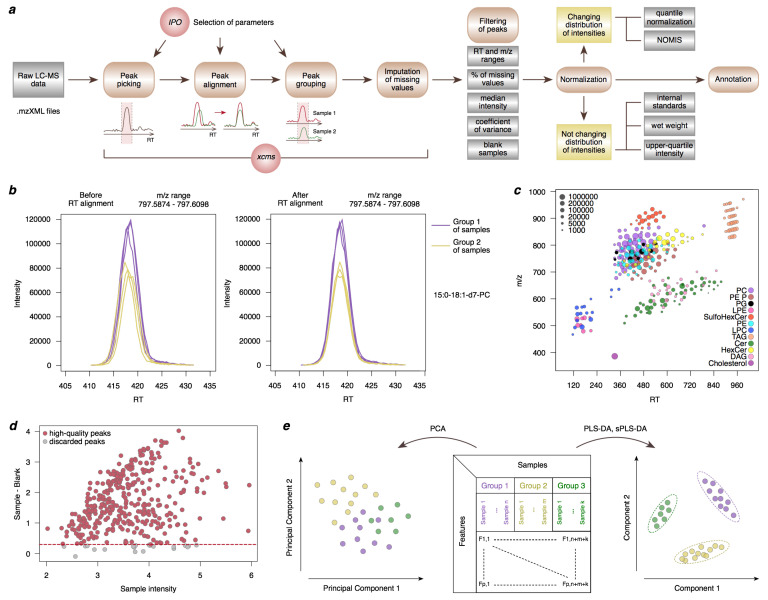
Schematic illustration of the LC-MS data analysis workflow. (**a**) Peak picking, alignment, and grouping are followed by the imputation of missing values, filtering, normalization, and annotation of lipid features. IPO and NOMIS abbreviations in the figure correspond to *IPO* [45,46] and *NOMIS* [48] tools, respectively. (**b**) An example of the peak alignment procedure for a deuterium-labeled lipid PC(15:0/18:1). (**c**) Mass and retention time of lipids with manually verified annotation based on a visually distinguishable ‘grid’ on this scatterplot. (**d**) A mean-difference plot visualizing the relationship of lipid intensities between biological samples and blank samples. For each peak, the median log10 intensities are calculated among biological samples and among blank samples. Each circle represents the sample intensity and the difference between the sample and blank intensities for a peak. The dashed red line shows the threshold of a two-fold difference between the sample and blank intensities used for peak filtering. (**e**) An illustrative example of Principal Component Analysis (PCA), Partial Least Squares-Discriminant Analysis (PLS-DA) and sparse PLS-DA score plots. Each data point on both plots corresponds to the coordinates of a single sample in a low-dimensional space.

Centroid data can be stored in multiple formats, depending on the MS instrument type. However, for further processing (Figure 2a), the files should be converted into a conventional mzXML format supported by most data analysis software, using the cross-platform *ProteoWizard* tool [43,44] or MS instrument vendor software.

### 3.2. Data Import

To give practical guidance, we illustrate the further steps of LC-MS data processing based on the *xcms* Bioconductor package (version 3.12.0) in the R environment [10,11,12], which is probably the most widely used solution among a multitude of available tools for MS data analysis.

However, before mzXML files can be imported into the R environment, they should be organized into a folder structure reasonable for the study design because *xcms* will guess the grouping of samples based on the subfolder structure and will align peaks between samples according to the folder hierarchy. Thus, the folder structure affects the grouping of peaks; the procedure matches MS peaks with similar m/z and RT across samples. mzXML files corresponding to samples that are expected to be most similar to each other (e.g., technical replicates) should be placed into a subfolder.

These subfolders should, in turn, be organized into higher-order hierarchies according to the study design and expectations about lipidome composition similarities between samples. Then, the data import can be performed with the *readMSData* command. In a detailed R notebook available at https://github.com/Khrameeva-Lab/lipidomics_analysis_2021 (accessed on 11 October 2021), we provide an example of the code that creates the list of files in the working directory, parses folder names to extract group labels for samples (i.e., mzXML files) stored in the folders, creates a metadata data frame, and finally, reads and imports all mzXML files.

### 3.3. Peak Picking

Untargeted LC-MS experiments aim to identify the abundances of individual lipid species characterized by unique m/z and RT values. To distinguish such peaks from background noise, a procedure of peak picking (i.e., MS peak detection) should be performed for all samples, with the *CentWaveParam* command setting the parameters for the peak picking procedure and *findChromPeaks* command performing peak picking for all samples. One of the most important parameters for these commands is *peakwidth* that defines the minimum and maximum possible MS peak width in RT dimension and can be adjusted based on ion chromatograms for internal standards, which can be extracted from the dataset using the *chromatogram* function. Another critical parameter is *ppm*, which defines the width of the region in the m/z dimension where all consecutive data points are combined before the peak detection procedure. It can be adjusted according to the mass accuracy of the employed LC-MS system.

### 3.4. Peak Alignment

Next, peaks identified at the previous step in each sample separately should be matched between samples. This is not a trivial task as chromatography can be affected by multiple factors leading to shifts in RT between measurement runs. Thus, the alignment procedure should be applied to adjust for these RT shifts from sample to sample (Figure 2b), with the *ObiwarpParam* command setting the parameters for the alignment procedure and *adjustRtime* command performing this procedure.

Of note, in this example, we use the Obiwarp algorithm [49], which is considered to be optimal for the untargeted LC-MS data. It is based on the dynamic time warping, which aims to make two samples as similar as possible via finding the best stretching of the time dimension [50]. The default parameters define the reference sample for the alignment as the one containing the largest number of peaks. The two most important parameters, *gapInit* and *gapExtend*, control the penalties in the warping optimization algorithm.

### 3.5. Peak Grouping

Finally, aligned peaks corresponding to the same lipid species should be grouped across samples. We illustrate this step using the PeakDensity algorithm [10], which iterates through the slices of m/z values and groups peaks according to the RT, as peaks representing the same lipid species are expected to cluster at the RT axis. Peak grouping can be performed using the *PeakDensityParam* command that sets the parameters for the peak grouping procedure and the *groupChromPeaks* command that performs peak grouping across all samples. The *minFraction* parameter defines the minimum proportion of samples in which a peak has to be detected.

This is where the folder structure of mzXML files becomes important because *xcms* calculates this proportion within a group of samples (i.e., within the lowest-hierarchy subfolder). The *minSamples* parameter works similarly, except it defines the minimum number of samples instead of the minimum proportion. The *binsize* parameter defines the width of the bin in the m/z dimension in which peaks are grouped. The *bw* defines the RT window used for the density function smoothing. Finally, the *maxFeatures* parameter limits the maximum number of features defined in one bin.

### 3.6. Selection of Parameters for Peak Picking, Alignment, and Grouping

In this workflow, we provide parameter settings optimized for untargeted lipidome LC-MS measurements on a Reversed-Phase Bridged Ethyl Hybrid (BEH) C8 column reverse coupled to a Vanguard precolumn, using a Waters Acquity UPLC system and a heated electrospray ionization source in combination with a Bruker Impact II QTOF mass spectrometer (Bruker Daltonics, Germany). However, in addition to the MS system vendors, the choice of parameters depends on multiple experimental conditions, such as the chromatographic separation buffers and gradient, MS settings, and the ion polarity mode.

Thus, the peak picking, alignment, and grouping parameters should be customized for the employed LC-MS system. One can start with the parameters recommended in the literature for a similar LC-MS system or with the default parameters for *findChromPeaks*, *adjustRtime*, and *groupChromPeaks* functions, and then manually adjust parameters one by one until the most appropriate settings are found. To visually inspect the outcomes of the parameter adjustment procedure, it is useful to plot a subset of well-known peaks (e.g., internal standards or known lipids) in the m/z versus RT coordinates (Figure 2c).

However, the manual choice of parameters is time-consuming and arbitrary. Therefore, we recommend optimizing *xcms* parameters using the Bioconductor package *IPO* [45,46]. First, *getDefaultXcmsSetStartingParams* and *getDefaultRetGroupStartingParams* commands set the range of possible parameter values for *IPO* to scan. Then, *optimizeXcmsSet* and *optimizeRetGroup* commands optimize peak picking, retention time correction, and grouping parameters within the specified ranges of possible parameter values. Finally, the *writeRScript* command returns the result of optimization in the form of an R script, which can be directly used to process raw mzXML files with *xcms*.

### 3.7. Imputation of Missing Values

Errors in the peak picking procedure frequently result in missing values, which can be imputed by the *fillChromPeaks* function integrating the signal that corresponds to the area of missing peak in the raw data. Of note, this procedure does not impute all missing values, while the absence of missing values is critical for downstream data analysis methods, such as Principal Component Analysis (PCA). Zero values not filled by the *xcms* imputation procedure can be further replaced using data-driven imputation techniques, such as Random Forest (RF), k-Nearest Neighbors (KNN), and Singular Value Decomposition (SVD) or simply by the limit of detection (LOD) value [51].

### 3.8. Data Export

Commands *chromPeaks*, *featureDefinitions*, and *featureValues* extract the data matrix, where the peak intensity is defined as the integral of the area under the peak. The last command produces a peak intensity matrix containing abundances of lipid species (rows) in all samples (columns).

### 3.9. Filtering of Peaks

MS peaks falsely duplicated during the *xcms* peak grouping procedure can be defined using a 10 ppm mass threshold (calculated as m/z difference divided by m/z and multiplied by 10${}^{6}$) and 1 s retention time difference. RT and m/z thresholds should be chosen to cover lipid classes of interest, e.g., from 1 to 18 min and from 120 to 1200 m/z in this example. In addition, peaks containing a high number of missing values are typically removed, as well as peaks with low median intensity and high variability in intensity calculated as the coefficient of variance (CoV), standard deviation (SD), or interquartile range (IQR).

As high-quality peaks typically have high variability among biological samples and low variability among technical replicates (e.g., pooled QC samples), CoV, SD, and IQR are usually calculated among pooled QC samples for each MS peak. A commonly used cut-off for filtering based on CoV is 25%. However, recent studies argue that CoV, SD, and IQR might be poor predictors of peak quality because they ignore biological variability [52]. The intra-class correlation coefficient (ICC) might be used instead as it simultaneously considers technical and biological variability.

To account for possible extraction and other technical contaminations, the concentrations in extraction blanks should be compared to the sample concentrations. MS peaks with less than a two-fold difference between the sample average and extraction blanks average should be discarded from the analysis. A mean-difference plot is a helpful way to visualize the relationship between the sample and extraction blank lipid abundances (Figure 2d) [52].

### 3.10. Normalization

Several data normalization approaches can be applied to lipidomics data. The most widely used ones operate by scaling all intensities in one sample by the same normalization factor (biomass, internal standard, mean, median, and sum intensity of features) and do not change the distribution of intensities. Typically, lipid intensities are normalized on either spiked-in internal standards representing most of the main lipid classes or the wet weight of the sample. Other normalization approaches change the distribution of intensities as each peak in each sample has its own normalization factor.

For instance, quantile normalization [53] stretches the distributions of all samples to make them similar, while the NOMIS approach [48] scales intensities by multiple internal standards, applying each standard to a corresponding range of RT values. However, a general assumption for all these normalization strategies is that most lipids are not affected by the factor of interest. If this is not the case, the best option would be to look into the raw data: if the desired effect is not visible in the raw data, it might be created by the normalization procedure and is not reliable.

In a specific case of experimental design with multiple biologically different samples from the same individual, the lipid intensities may be additionally normalized by the median abundance level within each individual to reduce individual-to-individual variability. To estimate the variability, it is useful to calculate the variance explained by each known covariate (e.g., sex, age, PMI, batch, individual, and others) using the *manova* function in R for all lipids using the following model: Y∼Sex+Age+PMI+Batch+Individual.

If sex, age, PMI, and other known covariates account for less variance each than the individual covariate, it suggests that there might be an additional hidden source of individual-to-individual variability as the order of covariates in the model is important for the calculation of the explained variance. Thus, we can transform our model into the following one: Y∼Individual+Sex+Age+PMI+Batch. If sex, age, RIN, and other known covariates account for a small proportion (e.g., less than 1%) of the variance in this model, while the individual covariate explains a substantial proportion of variance, the normalization by the median lipid abundance level within each individual is necessary and sufficient.

### 3.11. Annotation

The easiest way to annotate MS peaks is to match each peak with lipids from a predefined database allowing mass difference with peak m/z below the given threshold (e.g., 10 ppm). The lipid database can be downloaded from the Web (e.g., LIPIDMAPS [13], SwissLipids [54]) or constructed for specific lipid classes by varying the chain lengths and number of double bonds. All possible adducts—small ions that attach to or detach from lipid molecules under the ionization step (e.g., H${}^{+}$, Na${}^{+}$, and NH4${}^{+}$) and make them detectable by MS—should be considered.

Despite high precision, MS data frequently have a slight shift in the determined m/z-values. This shift can be found and consequently accounted for as a mode of distribution of directed annotation ppm values. For lipid classes with a sufficient number of detected members, a visually distinguishable ‘grid’ on the m/z versus RT scatterplot (Figure 2c) can be found that allows manual or semi-automatic filtering of MS peaks with RT not matching the grid-like pattern, additionally using internal standard RT as an anchor point when available. This manual filtration procedure is performed for positive and negative ionization modes depending on the lipid class. Finally, the ionization mode and adduct for which the lipid class has the highest relative intensities is used in further analysis.

Our annotation approach results in Level 3 identification (“putatively characterized compound classes”) according to the Metabolomics Standards Initiative guide [55]. Namely, all lipid species are determined on a ’tentative structure’ level relying on MS1 data exclusively. Proposed structures do not distinguish positional isomers (sn-attachment of fatty acids), carbon–carbon double bond positions (e.g., 18:2(n-6,n-9)) for unsaturated lipids and double bond geometry (cis- or trans-configurations). Proposed lipid annotations correspond to bulk lipid formulas (e.g., PE O-36:2) or ’bond type level’ [56] due to the high-resolution nature of MS measurements. Discrimination between ether-linked lipids (plasmanyl- and plasmenyl-species) may be performed by elution order on reversed-phase chromatographic systems.

## 4. Results

### 4.1. Visualization of LC-MS Data

LC-MS data analysis workflow results in normalized and annotated MS peaks, which can be further visualized. Lipid features are extremely different in amplitude and demonstrate heteroscedasticity—biological and technical variance are higher for features with high intensity. Thus, centering and scaling of intensities has to be performed prior to visualization as it equalizes the contributions of features to the separation of samples in multivariate space and makes the features comparable.

Lipid intensities can be scaled by the minimum and maximum values. However, this procedure is sensitive to outliers and is, thus, undesirable. Better approaches involve scaling by the standard deviation (SD) or by the root of SD (Pareto-scaling). The centering procedure is based on subtracting the mean or median intensity from all values. Finally, log transformation is typically applied because it has a scaling-like effect making features more comparable and helps to reveal multiplicative relations between features.

Principal Component Analysis (PCA) is a multivariate approach widely used to visualize lipidomics data, perform sample-level quality control, and explore differences in the lipidome profiles between sample groups [57]. The main objective of PCA is to project the original multivariate data to the low-dimensional space while preserving as much information about the original data as possible. A set of uncorrelated variables forming this new low-dimensional space is called Principal Components (PCs).

Principal components are ranked according to the proportion of variance explained in decreasing order so that PC1 always explains the most considerable variation of the original data. In the case of lipidomic data, new PCs represent vectors of the linear combination of original features. For a lipidome matrix, where features are in rows and samples are in columns, the set of PCs can be calculated using the *prcomp* function in R. Once PCs are calculated, one can proceed to the graphic representation of the method plotting the most informative PCs against each other (Figure 2e).

In this PCA plot, samples with similar lipidomic profiles tend to appear close together in a new reduced space, forming clusters. Thereby, it is possible to capture sample-specific differences between experimental conditions, assess group variances, and obtain an estimation of the data quality. The ability of PCA to identify outlier samples makes its application essential for the correct interpretation of conducted experiments prior to statistical analysis. Some noteworthy implementations of the PCA method in lipidomics studies include analyses of lipid profiles in drug-resistant prostate cancer [58], early Alzheimer’s disease [59,60], and coronary heart disease [61].

Partial Least-Squares Discriminant Analysis (PLS-DA) is a calibration algorithm that has become incredibly popular in the field of lipidomics [62,63,64,65]. In contrast with the classic PCA technique, PLS-DA can be considered as a “supervised” method and might be especially useful when dealing with a dataset for which a class membership for each sample is known. The general idea of PLS-DA is to project predictor variables and response variables to new low-dimensional space while preserving, in the first PLS component, as much covariance between them as possible.

A PLS-DA model in its standard variant can be constructed and subsequently visualized using *plsda* and *plotIndiv* functions from the *mixOmics* R package [47]. Lipid names, along with their scores of contribution into the first component, might be extracted from the model using the *selectVar* command. Of note, there is a sparse version of the PLS-DA method (sPLS-DA) that performs variable selection on a subset of all possible covariances [66,67].

While PLS-DA is widely accessible and may be helpful in many cases, it also has several drawbacks, e.g., the problem of overfitting or dependence on the distribution within sample classes [67,68,69,70,71]. Gromski et al. have investigated the efficiency of PLS-DA for the classification and feature selection problems and concluded that it has a rather low prediction accuracy for a small number of predictor variables compared to LDA, SVM, and RF-based approaches [71]. Therefore, one should be especially cautious when applying PLS-DA for the mass-spectrometry data analysis.

### 4.2. Applications of Untargeted Lipidomics

The main benefit of untargeted LC-MS approaches lies in their ability to measure many components simultaneously in complex lipid mixtures in an unbiased way. By contrast to shotgun lipidomics, which omits the chromatography step, untargeted LC-MS offers accurate separation and detection of lipids spanning a wide range of classes. Targeted LC-MS measurements are more sensitive, accurate, and quantitative than untargeted ones. Yet, they focus on particular lipid classes or species and are poorly suitable for descriptive studies aiming to generate hypotheses due to this detection bias. Thus, untargeted LC-MS analysis is the technology of the first choice for biomarker discovery studies because of the unbiased sample preparation and lipid detection, not favoring any particular lipid class [72].

The main limitation of untargeted LC-MS measurements is their semi-quantitative nature. Absolute quantification is challenging to achieve in LC-MS experiments as it requires extensive use of internal standards. The ion response within the lipid class can depend on the fatty acid composition, creating an additional complicating factor for absolute quantification [7]. However, for most experimental designs, the relative differences in lipid abundances are sufficient. For example, studies searching for biomarkers, i.e., changes in the lipidome composition between patients and controls or between knockout and wild-type samples, would result in a list of lipids showing statistically significant differences in concentrations between two sample groups of interest.

Absolute quantification of the lipid concentration is not needed to compose such lists. It is enough to accurately measure differences between sample groups, which is a feasible and suitable task for untargeted LC-MS. Standard statistical approaches, e.g., the Wilcoxon test with multiple testing correction, can be applied to the LC-MS data to find significant lipid abundance differences and detect potential biomarkers. To cautiously apply statistical methods and avoid possible mistakes in interpreting results, it is highly recommended to involve a biostatistician, especially at the study design stage, and for the final validation of applied statistical procedures. In addition, detected candidate biomarkers and lipid composition changes can be (and should be) further validated using targeted LC-MS or MS/MS approaches.

Another limitation of untargeted LC-MS approaches is the possible suppression of ionization caused by the complexity of lipid mixtures [7]. Thorough chromatographic separation prior to MS analysis helps to overcome this issue; however, this might not be practical for large-scale studies measuring the lipidome composition in thousands of samples because of the incredibly long time required to run the measurements.

### 4.3. Future Challenges

Thus, achieving high-quality chromatographic separation in a short run time is among the critical future challenges of LC-MS technology because this affects the scalability of lipidomics studies, which tend to analyze a large number of samples. Similar to Genome-Wide Association Studies (GWAS), increasing the number of analyzed samples is necessary to achieve the power to detect significant biomarkers in lipidomics studies where the expected effect size is relatively small. To keep such studies within reasonable time frames, either the chromatographic separation time should be reduced, or the number of MS machines should be increased to enable parallel runs. However, the last option dramatically increases experimental costs and introduces unwanted technical confounding factors and batch effects.

Even without parallel runs, batch effects constitute another future challenge of LC-MS technology. Small batch sizes are poorly suitable for large-scale lipidomics studies comprising thousands of samples because an accurate balancing of multiple confounding factors is difficult to achieve within a typical batch of 48–96 samples.

Apart from technological challenges, large-scale lipidomics studies introduce novel challenges at the data analysis level because they generate an extraordinary amount of data that must be stored, processed, and analyzed efficiently. The increased resolution of novel MS systems addresses to this problem as well as the need to measure the lipid composition in many technical or biological replicates to overcome technical or biological variability.

Limited databases and tools for the annotation of lipid species constitute another problem. Currently, most of them support only matching by m/z characteristic, without RT contribution, which depends on many technical factors and can only be used in in-house solutions for systems running with fixed parameters and stable environmental conditions.

The final and potentially most challenging problem resides in the lack of comprehensive curated lipid pathway databases linking lipids with proteins or genes. Multi-omics studies are in high demand but the few existing tools that are suitable for integrating different omics data types, i.e., lipidomics and transcriptomics, are mostly data-driven. Using correlations or more advanced metrics, they extract interrelationships of biomolecules from multi-omics data [73]. While such predicted links are of use for biomarker discovery, their biological interpretation is very limited, and curated biochemistry-based resources are essential for validation.

However, the Kyoto Encyclopedia of Genes and Genomes (KEGG) [74,75,76], REACTOME [77], and other widely used curated biochemical pathway databases cover only a limited set of lipid pathways, and mainly at the level of lipid classes but not individual lipid species. A detailed curated pathway database covering reactions of lipid species among all lipid classes would be an invaluable resource for the lipidomics community and future multi-omics studies.

## Data Availability

The R code performing the main steps of untargeted LC-MS data analysis described in this paper and a testing lipidome dataset is freely available at GitHub: https://github.com/Khrameeva-Lab/lipidomics_analysis_2021 (accessed on 11 October 2021).

## Supplementary Materials

The following are available online at https://www.mdpi.com/article/10.3390/metabo11110713/s1.
