# Supplementary material for: The Hitchhiker’s Guide to Untargeted Lipidomics Analysis: Practical Guidelines
Source: Metabolites. 2021 Oct 20;11(11):713. doi: 10.3390/metabo11110713 (PMC8624948; doi:10.3390/metabo11110713)
Supplement: Supplementary file 1 [file metabolites-11-00713-s001.zip › metabolites-1393960-supplementary.pdf]

Table S1.
A non-comprehensive list of tools suitable for the analysis of LC-MS data.

Tool	Implementation	Last update	Description
XCMS	Web, R	2021 (R version)	Performs all preprocessing steps for untargeted LC-MS data from data input to statistical testing and visualization.
LipidFinder	WEB, Python	2021	Identifies lipid features from pre-aligned data using information from LIPID MAPS (COMP_DB and LMSD databases). Additionally performs statistical analysis on identified lipids. Part of the LIPID MAPS web interface.
MZmine 2	Java	2019	Provides modules for raw data filtering, peak calling, peak processing and downstream analysis. Along with XCMS, MZmine 2 is the most widely used program for LC-MS data analysis.
MetaboAnalyst 5.0	Web, R	2021	Incorporates a large number of functions for downstream analysis, including statistical testing methods, enrichment and pathway analyses. Requires XCMS functionality for raw LC-MS spectra processing.
Galaxy-M	R, Python, MATLAB	2016	A pipeline for LC-MS metabolomics working in the Galaxy environment.
LipidXplorer	Windows executable	2019	Implements functionality for peaks alignment and subsequent lipid identification based on Molecular Fragmentation Query Language (MFQL) approach.
OpenMS	Linux, Windows, macOS executable	2021	“All-in-one” solution for the lipidomics data analysis. Includes *ThermoRawFileParser* data conversion.
Lipid Data Analyzer (LDA)	Java	2021	A tool for lipid species interpretation. It is also able to detect novel features.
LipidHunter 2	Python	2020	Identifies phospholipids, glycerolipids, and lysophospholipids. Supports multiple instrument vendors.

MetAlign 3	Windows executable	2018	Provides a large number of functions for MS data preprocessing (e.g., for baseline correction, peak picking, annotation)
LipidMatch	R	2020	Performs rule-based lipid identification within LC-HRMS/MS data.
LipidMS	R	2021	An R package developed for lipid annotation in LC-DIA-MS data.
LipidSearch (Thermo Scientific)	-	-	A commercial software suitable for raw LC-MS data processing and lipid identification using an internal library of ion masses.
MSClust	Windows executable	2012	Reduces the number of redundant peaks using the subtractive fuzzy clustering approach.
LipidBlast	-	2014	Provides a comprehensive database for peak annotation.
MS-DIAL 4	Linux, Windows, macOS executable	2020	Provides a graphical interface for peak identification within untargeted metabolomics data. Also holds modules for normalization and multivariate analysis. Supports multiple instruments, including LC-MS.
LIQUID	Windows executable, C# .Net	2021	Similar to MS-DIAL, it allows peak identification through a graphical interface, but is focused on lipids in LS-MS/MS data.
lipidr	R	2021	Provides a set of functions for downstream analysis of LC-MS lipidomics data.
lipyd	Python	2020	Provides workflow for lipidomics data analysis. Requires OpenMS for the data preprocessing step.
LipiDex	Java	2018	Allows gaussian peak modeling based lipid identification and result filtering.
LION	Web	2020	Provides functionality for the enrichment analysis of lipids using a GO database containing information about lipid classification, subcellular localization, and biophysical properties.

LICRE 1.0	MATLAB	2017	Reduces the number of redundant features in lipidomic data by identification and pruning of groups of highly correlated lipids.
LipidSig	Web	2021	Implements a web-based workflow for downstream analysis of lipidomics data.
LipidLynxX	Windows executable, Web	2020	Provides resources for lipid annotation and ID conversion. Part of the LIPID MAPS web interface.
LPPtiger	Windows executable	2017	Performs detection of oxidized phospholipids.
Cytoscape 3	Cross-platform	2021	Allows network analysis and visualization of omics datasets, including lipidomics profiles.
LIPEA	Web	2018	Performs Over Representation analysis of lipid features using KEGG pathways.
